# HO-1 nuclear accumulation and interaction with NPM1 protect against stress-induced endothelial senescence independent of its enzymatic activity

**DOI:** 10.1038/s41419-021-04035-6

**Published:** 2021-07-26

**Authors:** Wenwei Luo, Jingyan Li, Ziqing Li, Tong Lin, Lili Zhang, Wanqi Yang, Yanqi Mai, Ruiming Liu, Meiting Chen, Chunmei Dai, Hanwei Yang, Jing Lu, Hong Li, Guimei Guan, Min Huang, Peiqing Liu, Zhuoming Li

**Affiliations:** 1 Department of Pharmacology and Toxicology, School of Pharmaceutical Sciences, National and Local United Engineering Lab of Druggability and New Drugs Evaluation, Guangdong Engineering Laboratory of Druggability and New Drug Evaluation, Guangdong Provincial Key Laboratory of New Drug Design and Evaluation, Sun Yat-sen University, Guangzhou, China; 2 International Institute for Translational Chinese Medicine, School of Pharmaceutical Sciences, Guangzhou University of Chinese Medicine, Guangzhou, China; 3 Department of Vascular and Surgery, The First Affiliated Hospital, Sun Yat-sen University, Guangzhou, China; 4 Emergency Department, The Second Affiliated Hospital of Guangzhou Medical University, Guangzhou, China; 5 Department of Biochemistry and Molecular Biology, Guangzhou University of Chinese Medicine, Guangzhou, China; 6 Department of Obstetrics and Gynecology, The First Affiliated Hospital, Sun Yat-sen University, Guangzhou, China; 7 Institute of Clinical Pharmacology and Guangdong Provincial Key Laboratory of New Drug Design and Evaluation, School of Pharmaceutical Sciences, Sun Yat-sen University, Guangzhou, China

**Keywords:** Stress signalling, Vascular diseases

## Abstract

Heme oxygenase-1 (HO-1) has attracted accumulating attention for its antioxidant enzymatic activity. However, the exact regulatory role of its non-enzymatic activity in the cardiovascular system remains unaddressed. Here, we show that HO-1 was accumulated in the nuclei of stress-induced senescent endothelial cells, and conferred protection against endothelial senescence independent of its enzymatic activity. Overexpression of ΔHO-1, a truncated HO-1 without transmembrane segment (TMS), inhibited H_2_O_2_-induced endothelial senescence. Overexpression of ΔHO-1_H25A_, the catalytically inactive form of ΔHO-1, also exhibited anti-senescent effect. In addition, infection of recombinant adenovirus encoding ΔHO-1 with three nuclear localization sequences (NLS), alleviated endothelial senescence induced by knockdown of endogenous HO-1 by CRISPR/Cas9. Moreover, repression of HO-1 nuclear translocation by silencing of signal peptide peptidase (SPP), which is responsible for enzymatic cleavage of the TMS of HO-1, exacerbated endothelial senescence. Mechanistically, nuclear HO-1 interacted with NPM1 N-terminal portion, prevented NPM1 translocation from nucleolus to nucleoplasm, thus disrupted NPM1/p53/MDM2 interactions and inhibited p53 activation by NPM1, finally resisted endothelial senescence. This study provides a novel understanding of HO-1 as a promising therapeutic strategy for vascular senescence-related cardiovascular diseases.

## Introduction

Endothelium, the single layer of cells lining the entire circulatory system, plays a pivotal role in maintaining vascular homeostasis [1]. Endothelial cells are vulnerable to cardiovascular risk factors such as oxidative stress, shear stress, hypertension, hyperlipidemia and hyperglycemia, and present a premature senescent phenotype [2–5]. Senescent endothelial cells are characterized by cell-cycle arrest, pro-adhesion, pro-inflammation and pro-thrombosis. Endothelial senescence remains an important part of vascular aging contributing to the initiation and progression of cardiovascular diseases including atherosclerosis, hypertension, and heart failure [6–8]. Therefore, strategies against endothelial senescence might suggest therapeutic potential for the treatment of cardiovascular diseases.

Accumulating evidences support that heme oxygenase-1 (HO-1) inhibits stress-induced endothelial senescence and improves endothelial function [9–11]. HO-1 is the rate-limiting enzyme in the degradation of heme to release free iron, biliverdin and carbon monoxide, and is regarded to be the most important endogenous protective enzyme against oxidative stress [12, 13]. Interestingly, our previous findings indicated that HO-1 ameliorated endothelial senescence through both enzymatic activity-dependent and -independent mechanisms [9]. Independent from its antioxidant activity, HO-1 interacted with endothelial nitric oxide synthase (eNOS), promoting the interaction of eNOS and Akt, thus enhancing eNOS phosphorylation at Ser1177 by Akt, subsequently increasing nitric oxide production [9]. These findings implicate that HO-1 might have unrecognized cellular functions beyond its enzymatic activity-dependent antioxidant effects.

Preliminary observations in the present study demonstrated that HO-1 can translocate into the nucleus in the senescent endothelial cell model induced by hydrogen peroxide (H_2_O_2_). Normally, HO-1 is a cytoplasmic protein which is anchored in the endoplasmic reticulum (ER) through a carboxyl-terminal single transmembrane segment (TMS) [14, 15]. Nuclear localization of HO-1 in endothelial cells has not been reported, and the functions of nuclear HO-1 remain unclear. In view of this, the present study attempted to investigate the possible mechanisms underlying HO-1 nuclear accumulation, and to clarify the effects of nuclear HO-1 in regulating vascular endothelial senescence.

## Materials and methods

### Cell culture

Neonatal umbilical cords from were collected from healthy puerperants undergoing cesarean operation in The First Affiliated Hospital, Sun Yat-sen University. Human umbilical vein endothelial cells (HUVECs) were primary-cultured as described previously [9]. HUVECs were grown in endothelial cell specific medium with supplement mix (ECM, ScienCell, USA). Several cellular senescence models were built: (1) HUVECs were stimulated with 50 μM hydrogen peroxide (H_2_O_2_, Calbiochem, USA) for 1 h, and then medium was replaced 1 h later and the culture was continued further for 48 h. (2) HUVECs were stimulated with 1 μM Ang II (Sigma-Aldrich, St. Louis., MO, USA) for 48 h and supplemented once every 24 h. (3) HUVECs were stimulated with 50 mM D-gal (Sigma-Aldrich, St. Louis., MO, USA) for 48 h. (4) HUVECs were stimulated with 100 μg/mL oxLDL (Yiyuan Biotechnologies, Guangzhou, China) for 2 days. (5) HUVECs were sub-cultured to the 12th generation as a replicative senescence model.

### Animals

Animal procedures used in this study were in accord with institutional guidelines and were approved by Laboratory Animal Center of the Sun Yat-sen University. Two-month-old and 23-month-old male C57BL/6 J mice were purchased from Jiangsu ALF Biotechnology Co., LTD. All the mice used in this study were chosen randomly. The mice were euthanized and perfused with 0.1 M KCl solution via the left ventricle, and then aortas were isolated and removed of periadventitial tissues collected and prepared for frozen sections subjected to staining.

### Mice model with partial carotid ligation

Male C57BL/6 J mice were ligated between 8 and 10 weeks of age. Partial ligation of left common carotid artery (LCA) was performed as reported [16]. Briefly, the anesthetized mice were made a ventral midline incision (4–5 mm) in the neck. Three caudal branches (external carotid, internal carotid, and occipital artery) of LCA were ligated with 6-0 silk sutures (Fig. S1A). Ultrasound measurements were taken using a VEVO 3100 high-resolution in vivo microimaging ultrasound system. 48 h later, LCA and right common carotid artery (RCA) were isolated and collected for the preparation of frozen sections subjected to staining.

### RNA interference

Negative control small interfering RNA (NC-siRNA) and knockdown experiments siRNA were purchased from Guangzhou Ribobio Co., Ltd. Sequences of siRNA targeting signal peptide peptidase (SPP) were as follows: siRNA-1: ATATTCTCCCAGGAGTACA, siRNA-2: TCTTCGTGCTGGGAATCCT, siRNA-3: GGACTCGGCCCTCAGCGAT. HUVECs were transfected with 20 nM SPP-siRNA or with NC-siRNA in Opti-MEM (Gibco, Grand Island, NY, USA), using Lipofectamine 2000 (Invitrogen, Groningen, Netherlands). The medium was removed and replaced with fresh ECM after 5 h, and the cells were maintained for 48 h before further experiments.

### CRISPR/Cas9 sgRNA knockdown

CRISPR/Cas9 sgRNA knockdown lentiviral vectors (GV392: lentivirus-sgRNA-Cas9-puromycin) were constructed by Shanghai Genechem Co., Ltd. Sequences of sgRNA targeting HO-1 were as follows: sgRNA-1: CCGCTTCACATAGCGCTGCA, sgRNA-2: AAGGGCCAGGTGACCCGAGA, sgRNA-3: GAACTCAGCATTCTCTGCCT. HUVECs were cultured in plates until 60% confluence, and then infected with HO-1-sgRNA lentivirus. Medium was changed to fresh ECM after 6 h and HUVECs were cultured for another 72 h. HO-1 knockdown cells were obtained via the screening of puromycin, and the deficient efficiency of HO-1 was tested by Western blot.

### Plasmid transfection

HO-1 truncated plasmid with a Flag tag (without TMS sequence, ΔHO-1, amino acids from 1 to 266/aa1-266) and point mutation plasmid (catalytically inactive, ΔHO-1_H25A_) were constructed by Shanghai Generay Co., Ltd. HO-1 truncated plasmid with a Flag tag (aa1–65; 66–130; 131–266) and NPM1 wild type plasmid (with a Flag tag) were constructed by Shanghai Sangon Biotech Co., Ltd. HUVECs were incubated with 2 μg/mL plasmid and DNA transfection reagent jetOPTIMUS (Polyplus, France) in Opti-MEM. After 5 h of transfection, the medium was changed, and the cells were sequentially cultured for 2 days.

### Adenovirus infection

Recombinant adenovirus encoding truncated HO-1 with 3 nuclear localization sequences (NLS: KRPAATKKAGQAKKKK ×3) were constructed by Shanghai Genechem Co., Ltd. To avoid the interference of HO-1-sgRNA, exogenous ΔHO-1 were synonymous mutated. HUVECs were incubated with recombinant adenovirus Ad-ΔHO-1(3NLS) or Ad-Vector at 10 MOI for 6 h and then cultured in fresh ECM for 48 h.

### Western blot

After treatments, the cells were harvested and lysed with cell lysis solution. The protein concentration was determined by BCA Protein Assay Kit (Pierce, Rockford, IL, USA). The nuclear proteins were extracted by Nuclear Extract Kit (Active Motif, USA) and determined by Bradford method. Equal amounts (15–30 μg) of protein were subjected to SDS gel electrophoresis (8–15% gel) for ~90 min at 120 V and transferred to PVDF membrane by wet electro-blotting (230 mA, 120 min) using the standard Western blot protocol. Immune-reactive protein signals were visualized by enhanced chemiluminescence detection (ECL+, Tanon Shanghai, China). The protein bands were quantified with Image J 1.40 g software. The following antibodies were used: rabbit polyclonal anti-HO-1 (Proteintech, Chicago, USA, 10701-1-AP), mouse monoclonal anti-HO-1 (Abcam, Cambridge, UK, ab13248), rabbit monoclonal anti-HO-1 (Abcam, ab52947), rabbit polyclonal anti-SPP (Abcam, ab190253), rabbit polyclonal anti-α-Tubulin (Proteintech, 11224-1-AP), mouse monoclonal anti-Lamin B1 (Proteintech, 66095-1-Ig), rabbit polyclonal anti-GAPDH (Proteintech, 10494-1-AP), rabbit polyclonal anti-Histone H3 (Proteintech, 17168-1-AP), rabbit polyclonal anti-p53 (Proteintech, 10442-1-AP), mouse monoclonal anti-p53 (Proteintech, 60283-2-Ig), mouse monoclonal anti-p53 (Cell Signaling Technology, Boston, MA, #2524), rabbit polyclonal anti-p21 (Proteintech, 10355-1-AP), mouse monoclonal anti-NPM1 (Proteintech, 60096-1-Ig), rabbit polyclonal anti-fibrillarin (Proteintech, 16021-1-AP), rabbit polyclonal anti-GST (Proteintech, 10000-0-AP), rabbit polyclonal Flag (Proteintech, 20543-1-AP), mouse monoclonal anti-MDM2 (Santa Cruz Biotechnology, Santa Cruz, CA, USA, sc-5304), rabbit monoclonal anti-MDM2 (Cell Signaling Technology, MA, USA, #86934), secondary polyclonal antibodies (Cell Signaling Technology, #7074, #7076).

### Real-time PCR

Total RNA of cells was extracted using RNAiso Plus reagent (TaKaRa, Tokyo, Japan) and was converted into cDNA by Revert Aid Kit according to the manufacturer’s instructions (Thermo Fisher Scientific, Waltham, MA, USA). Quantitative real-time PCR with 2^-ΔΔCT^ method was used to measure the relative levels of gene expression. The Following primer pairs (Sangon Biotech, Shanghai, China) were used: SPP, 5-ACCAGCTTTGCAGCCTACAT-3 (Forward) and 5-GGATTTGACTCCTCATAACTGAACA-3 (Reverse); GAPDH, 5-GGATTTGGTCGTATTGGG-3 (Forward) and 5-GGAAGATGGTGATGGGATT-3 (Reverse).

### Immunofluorescence

HUVECs were fixed with 4% paraformaldehyde for 30 min, washed with PBS for 5 min, and permeabilized in 0.1% Triton X-100 at room temperature for 10 min. The cells were incubated with rabbit polyclonal antibody against HO-1 (1:100), mouse monoclonal antibody against NPM1 (1:100), rabbit polyclonal antibody against fibrillarin (1:100) or mouse monoclonal antibody against p53 (1:50, Cell Signaling Technology, #2524) at 4 °C overnight. The cells were then washed with PBS and incubated with appropriate secondary antibodies in goat serum (Boster Biological Technology, California, USA) for 1 h at room temperature. Alexa Fluor 594-conjugated anti-rabbit IgG (H+L) secondary antibody and Alexa Fluor 488-conjugated anti-mouse IgG (H+L) secondary antibody (Proteintech, SA00013-4, SA00013-1) were used. Nuclei were stained with 5 μg/ml DAPI (Sigma). Images were acquired using cell auto imaging system (EVOS FL Auto, Life Technologies) or FV3000 laser scanning confocal microscope (Olympus Life Science).

### Cell cycle analysis

HUVECs were deprived serum for 24 h to synchronize cells in G0/G1 phase. Cells detached using Trypsin-EDTA (Gibco) were fixed with cold 70% ethanol overnight at 4 °C. The labeled cells were stained with PI/RNase Staining Buffer (BD Pharmingen, San Diego, CA, USA). Cell cycle analysis was performed using Guava easyCyte (Merck Millipore, Billerica, MA, USA) or Cytomics FC 500 flow cytometer (Beckman Coulter, Miami, FL, USA). The ratio of G0/G1 phase cells was counted by Tree Star FlowJo X 10.0.7 software.

### Senescence-associated β-galactosidase (SA-β-gal) staining assay

To evaluate endothelial cell senescence, SA-β-gal (Beyotime Biotechnology, Shanghai, China) staining was performed according to the instruction. Briefly, HUVECs or arterial sections were incubated with freshly prepared β-gal staining solutions for 12 h at 37 °C. Stained cells were imaged under a bright-field microscope at a magnification of 200×. SA-β-gal positive and total cells were counted, and the percentage of senescent cells was calculated.

### Cell proliferation arrest detection

HUVECs were cultured in M199 medium (Gibco) containing 50 μM 5-ethynyl-2’-deoxyuridine (EdU) for 2 h at 37 °C. Detection of EdU signal was achieved with the Cell-Light EdU Apollo 567 (RiboBio), according to the manufacturer’s protocol. The results were quantified with cell auto imaging system.

### RNA sequence analysis

HUVECs were transfected with vector or ΔHO-1 plasmid and treated with Hemin or not for 48 h before RNA extraction. RNA samples (3 samples per group) were analysed according to BGISEQ-500 platform by BGI-Shenzhen Co., Ltd. To be brief, the purifying mRNA was fragmented into small pieces and enriched with RT-PCR. PCR products were quantified and made a single strand DNA circle (ssDNA circle). DNA nanoballs were generated with the ssDNA circle by rolling circle replication to enlarge the fluorescent signals, and loaded into the patterned nanoarrays later. In total, 50 bp single-end read were read through on the BGISEQ-500 platform for the following analysis. Differently expressed genes (DEGs) was classified the into biological pathways, and anaylased by the phyper function in R software for the Kyoto encyclopedia of genes and genomes (*KEGG)* and gene ontology (*GO*) enrichment analysis. The raw RNA-seq data was uploaded to NCBI SRA database (SRA accession number: PRJNA729606).

### Immunoprecipitation-mass spectrometry (IP-MS) analysis and co-immunoprecipitation (co-IP)

Extracted nuclear proteins were used for HO-1 specific immunoprecipitation. HO-1 antibody was added to the cell lysates and incubated with rotation overnight at 4 °C. Mixed solution was incubated with protein A/G beads (Thermo Fisher) for 4 h at 4 °C. Then the beads were washed 3 times with washing buffer containing 150-500 mM NaCl. Lastly, the samples were washed 3 times with lysis buffer and then denatured by the addition of 2× loading buffer, boiled 5 min and analysed by mass spectrometry. Mass spectrometry analysis was accomplished by Shanghai BioClouds Co., Ltd. For co-immunoprecipitation assay, immunoprecipitation proteins were enriched by relevant primary antibodies and then analysed by Western blot. The following immunoprecipitation antibodies were used: rabbit polyclonal anti-HO-1 (1:100, Proteintech), mouse monoclonal anti-p53 (1:100, Proteintech), mouse monoclonal anti-NPM1 (1:100, Proteintech).

### GST pull-down

NPM1 wild type plasmid, point mutation plasmid (C275S) and truncated plasmids with GST tag of NPM1 (aa1-119; 120–188; 189–294) were constructed by Shanghai Generay Co., Ltd. Truncated plasmids with GST tag of HO-1 (aa1–65; 66–130; 131–266) were constructed by Shanghai Sangon Biotech Co., Ltd. Fusion proteins expressed by *E.coli* BL21(DE3) were respectively purified by GST Tag Immunomagnetic Beads (Sino Biological Inc., Beijing, China). HO-1 protein induced by Hemin or NPM1 protein overexpressed by plasmid was incubated with GST-beads for 1 h at 37 °C. The beads were washed 3 times with washing buffer and then denatured by the addition of 2× loading buffer, boiled 5 min and analysed by Western blot.

### Protein-protein docking

Single chain structure of HO-1 and NPM1 were obtained with PyMol software. Amino acid residues sequence of HO-1 (aa10–224) derived from 1ni6.pdb, and NPM1 (aa14–119) derived from 5ehd.pdb. HO-1-NPM1 docking was analysed by HADDOCK website (https://bianca.science.uu.nl/haddock2.4/). Interface residues of the best HO-1-NPM1 complex were analysed by PDBePISA website (https://www.ebi.ac.uk/msd-srv/prot_int/pistart.html).

### Statistical analysis

Data are presented as mean ± standard error of the mean (SEM), and analyzed by two-tailed unpaired Student’s t-test between two groups and by one-way ANOVA followed by the Bonferroni post hoc test for multiple comparisons using GraphPad Prism Software Version 8.0.2 (La Jolla, CA). *P* < 0.05 was considered to be statistically significant.

## Results

### Nuclear accumulation of HO-1 occurred in stress-induced senescent endothelial cells

**Fig. 1:**
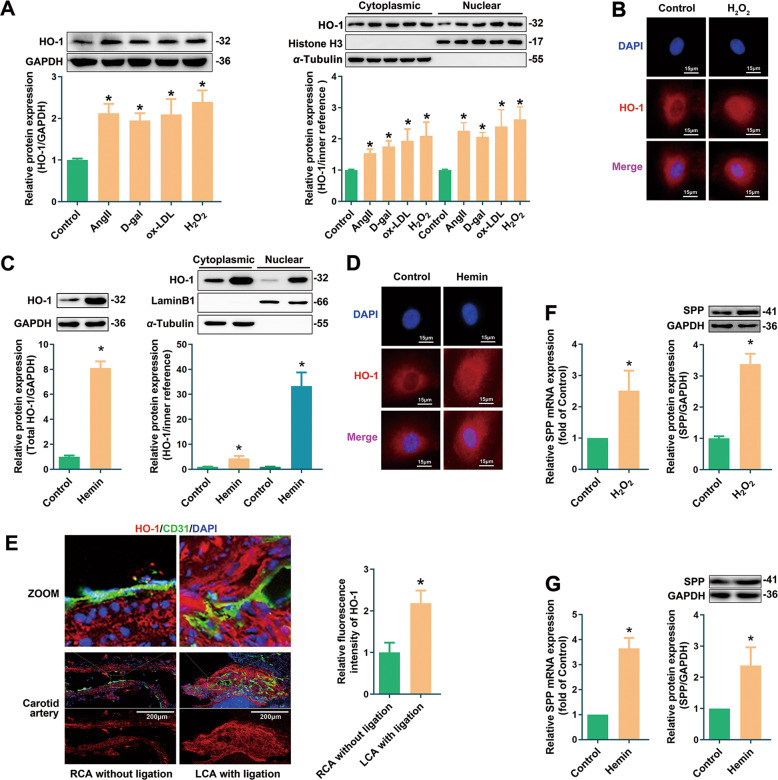
Nuclear HO-1 was up-regulated in prematurely senescent endothelial cells with SPP activation. **A** Upregulation of total, cytoplasmic and nuclear HO-1 expression in HUVECs induced by Ang II, D-gal, ox-LDL and H_2_O_2_. ^*^P <0.05 vs. Control. *n* = 5. **B** Immunofluorescence experiment showed that HO-1 was enriched in the nucleus of senescent endothelial cell induced by H_2_O_2_. *n* = 3. **C** Hemin increased the expression of total, cytoplasmic and nuclear HO-1. ^*^P < 0.05 vs. Control. *n* = 5. **D** HO-1 accumulation occurred in the cytoplasm and nucleus induced by Hemin. *n* = 3. **E** The level of HO-1 increased in LCA with ligation, ^*^P < 0.05 vs. RCA without ligation. *n* = 4. **F** Upregulation of SPP mRNA and protein level in HUVECs induced by H_2_O_2_. ^*^P < 0.05 vs. Control. *n* = 5. **G** Hemin increased the mRNA and protein level of SPP. ^*^P < 0.05 vs. Control. *n* = 5.

Endothelial senescence could be induced by several cardiovascular risk factors including H_2_O_2_, angiotensin II (Ang II), oxidized low density lipoprotein (ox-LDL) and D-galactose (D-gal) [5, 17]. In these stress-induced endothelial senescence models, the protein expression of HO-1 was increased (Fig. 1A). Surprisingly, the expression of HO-1 in the nuclear fraction, in addition to that in the cytoplasmic fraction, was significantly augmented (Fig. 1A). The immunofluorescence results demonstrated that HO-1 accumulation in nucleus was enhanced in HUVECs treated with H_2_O_2_ (Fig. 1B). In addition, we further investigated the cellular localization of HO-1 in endothelial cells treated with Hemin, a pharmacological inducer of HO-1 which could ameliorate H_2_O_2_-induced endothelial senescence [9]. Similar as the observations in stress-induced senescent HUVECs, the level of nuclear HO-1 was enhanced by Hemin (Fig. 1C, D).

In vivo, the expression of HO-1 was measured in an ischemic stress-induced endothelial senescence model using mice with partial ligation of left carotid artery (LCA) [16]. In this model, blood flow was disturbed in LCA, and expression of senescence-associated-β-Galactosidase (SA-β-gal) was augmented in the endothelium of LCA with ligation, confirming that the endothelial cells in LCA presented a senescent phenotype (Fig. S1). The expression of HO-1 was upregulated in the endothelium of the ligated LCA (Fig. 1E), in line with the in vitro observations that HO-1 was upregulated in stress-induced endothelial senescence.

However, HO-1 was observed a decrease in replicative endothelial senescence induced by population doublings in cell culture, as well as in the senescent endothelium of aortas from 23 months old mice (Fig. S2). The nuclear expression of HO-1 was downregulated as well (Fig. S2).

It is still unclear why HO-1 was accumulated in nucleus of stress-induced senescent cells or Hemin-treated cells. HO-1 is anchored in ER, unless it is cleavaged by signal peptide peptidase (SPP), an ER-associated aspartyl protease catalyzing the proteolytic cleavage of HO-1 on its carboxyl-terminal TMS [15]. To test the hypothesis that upregulation of SPP facilitates the nuclear translocation of HO-1, the expression of SPP was assessed in HUVECs treated with H_2_O_2_ or Hemin. As indicated by Fig. 1F and G, SPP was upregulated at both the transcription and translation levels by H_2_O_2_ or Hemin, suggesting that increased expression of SPP may be associated with HO-1 nuclear localization in endothelial senescence.

### Overexpression of nuclear HO-1 resisted endothelial senescence

**Fig. 2:**
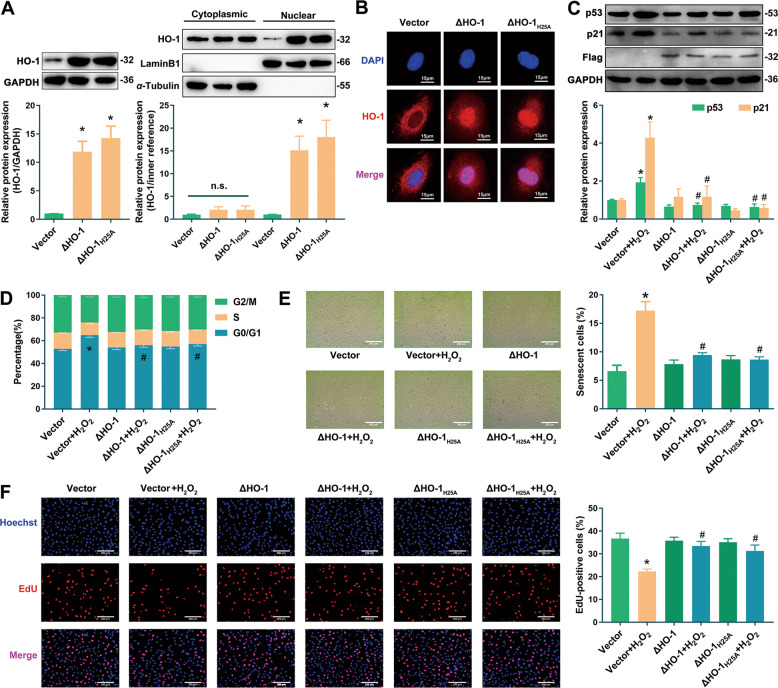
Overexpressing nuclear HO-1 by transfecting with ΔHO-1 or ΔHO-1_H25A_ resisted endothelial senescence. **A** Overexpression of HO-1 was detected in HUVECs transfected with ΔHO-1 or ΔHO-1_H25A_. ^*^P < 0.05 vs. Vector. *n* = 5. **B** Fluorescence signal of HO-1 was enriched in the nucleus. *n* = 3. **C** Nuclear accumulation of HO-1 in HUVECs transfected with ΔHO-1/ΔHO-1_H25A_ reversed the increasing expression of p53/p21 in senescent cells induced by H_2_O_2_. ^*^P < 0.05 vs. Vector; and ^#^*P* < 0.05 vs. Vector+H_2_O_2_. *n* = 4. **D** Nuclear accumulation of HO-1 ameliorated G0/G1 cell cycle arrest induced by H_2_O_2_. ^*^P < 0.05 vs. Vector; and ^#^*P* < 0.05 vs. Vector+H_2_O_2_. *n* = 5. **E** Nuclear accumulation of HO-1 reversed the increasing proportion of SA-β-gal-positive cells induced by H_2_O_2_. ^*^P < 0.05 vs. Vector; and ^#^*P* < 0.05 vs. Vector+H_2_O_2_. *n* = 5. **F** Nuclear accumulation of HO-1 reversed the decreasing ratio of EdU-positive cells. ^*^P < 0.05 vs. Vector; and ^#^*P* < 0.05 vs. Vector+H_2_O_2_. *n* = 4.

In order to explore the regulatory role of nuclear HO-1 in endothelial senescence, and to investigate the possible involvement of HO-1 enzymatic activity in the effect of nuclear HO-1, an HO-1 truncated plasmid without TMS sequence (ΔHO-1, with a Flag tag) and a catalytically inactive plasmid of ΔHO-1 (ΔHO-1_H25A_) [15] were constructed. Western blot (Fig. 2A) and immunofluorescence (Fig. 2B) analyses confirmed that transfection with ΔHO-1 or ΔHO-1_H25A_ in HUVECs increased the nuclear expression and distribution of HO-1 without altering its cytoplasmic expression. Since endothelial senescence is characterized by the up-regulation of cyclin-dependent kinase inhibitors such as p53 and p21, high-expression of SA-β-gal, cell cycle arrest and proliferation inhibition [18], the effects of ΔHO-1 and ΔHO-1_H25A_ on these senescent indicators were investigated. In the H_2_O_2_-induced endothelial senescence model, overexpression of ΔHO-1 or ΔHO-1_H25A_ reversed the up-regulation of p53/p21 (Fig. 2C), the increasing percentage of cells at the G0/G1 phase (Fig. 2D), the increasing ratio of SA-β-gal-positive cells (Fig. 2E) and the decreasing proportion of EdU-positive proliferative cells (Fig. 2F). These results suggest that upregulation of nuclear HO-1 resists endothelial senescence independent of its enzymatic activity.

**Fig. 3:**
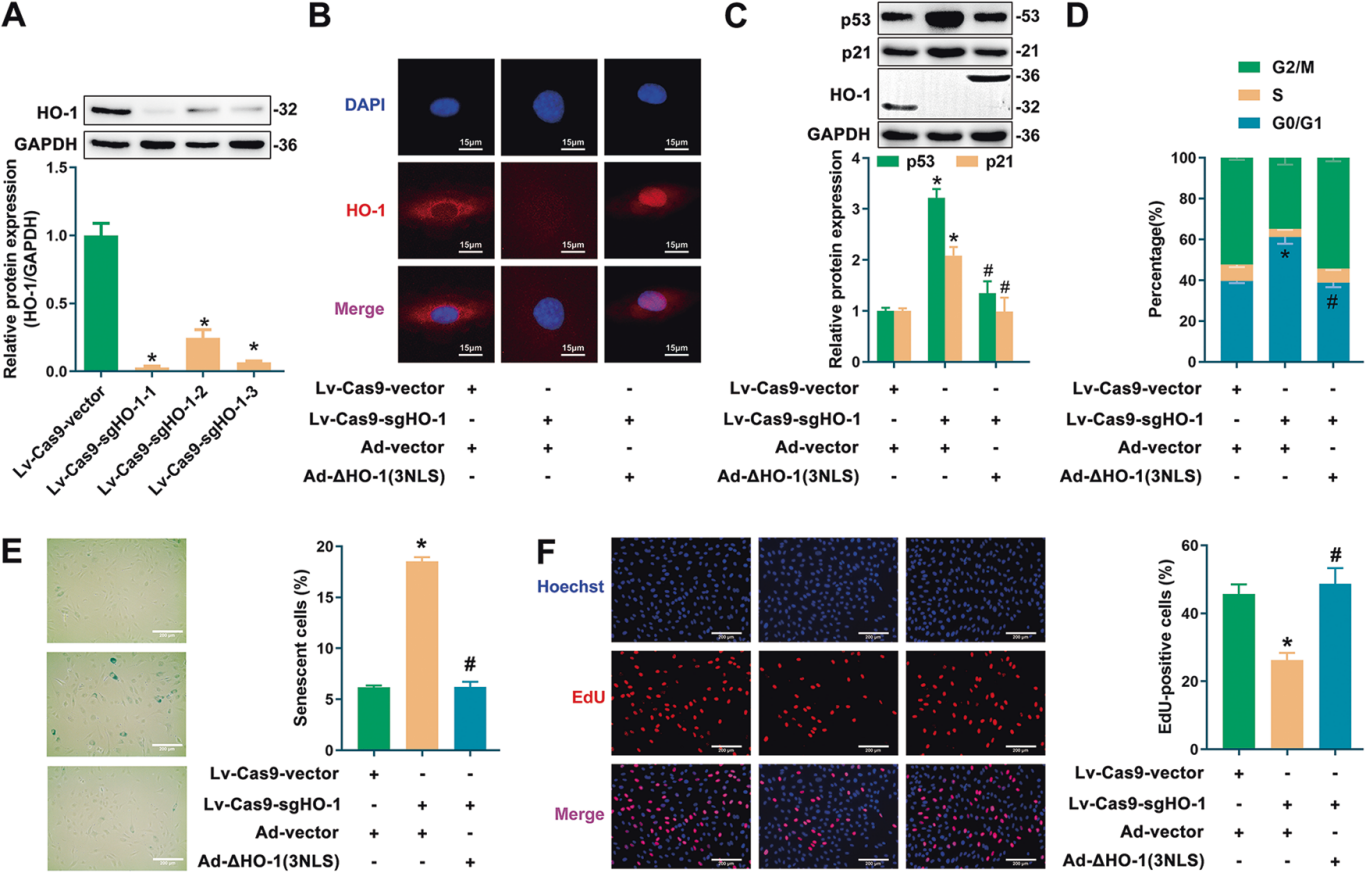
Overexpressing nuclear HO-1 after knockdown of endogenous HO-1 resisted endothelial senescence. **A** Deficient efficiency of sgRNAs was tested. ^*^P < 0.05 vs. Lv-Cas9-Vector. *n* = 5. **B** Fluorescence signal of HO-1 was detected. *n* = 3. **C** HO-1 knockdown by CRISPR-Cas9 increased the expression of p53/p21, but was reversed by Ad-HO-1(3NLS). ^*^P < 0.05 vs. vector; and ^#^*P* < 0.05 vs. Lv-Cas9-sgHO-1. *n* = 3. **D** Infection with Ad-HO-1(3NLS) ameliorated G0/G1 cell cycle arrest induced by HO-1 knockdown. ^*^P < 0.05 vs. vector; and ^#^*P* < 0.05 vs. Lv-Cas9-sgHO-1. *n* = 5. **E** Infection with Ad-HO-1(3NLS) reversed the increasing proportion of SA-β-gal-positive cells induced by HO-1 knockdown. ^*^P < 0.05 vs. vector; and ^#^*P* < 0.05 vs. Lv-Cas9-sgHO-1. *n* = 5. **F** Infection with Ad-HO-1(3NLS) reversed the decreasing ratio of EdU-positive cells induced by HO-1 knockdown. ^*^P < 0.05 vs. vector; and ^#^*P* < 0.05 vs. Lv-Cas9-sgHO-1. *n* = 5.

To further eliminate the interference of cytoplasmic HO-1 and to verify the sole role of nuclear HO-1 in regulating endothelial senescence, HO-1 was knocked down by CRISPR/Cas9, followed by the infection of recombinant adenovirus encoding truncated HO-1 without TMS but with three nuclear localization sequences (NLS: KRPAATKKAGQAKKKK ×3). As shown in Fig. 3A, the efficiency of three CRISPR/Cas9 sgRNA sequences targeting HO-1 were tested, and sgRNA-1 was picked up as the most efficient one for knockdown of endogenous HO-1. As indicated by the fluorescence staining experiments, fluorescent signals of endogenous HO-1 were disappeared in cells infected with HO-1-sgRNA lentivirus, while signals of ΔHO-1(3NLS) adenovirus were enhanced remarkably in nucleus (Fig. 3B). As compared with the control group, HUVECs knocked down endogenous HO-1 by Lv-Cas9-sgHO-1 presented senescent phenotype, characterized by the increase of p53/p21 expression, G0/G1 phase cells and SA-β-gal-positive cells, and the decrease of proportion of proliferative cells (Fig. 3C–F). By contrast, upregulation of nuclear HO-1 by infection with Ad-ΔHO-1(3NLS) prevented endothelial senescence induced by HO-1 knockdown, confirming the anti-senescent effect of nuclear HO-1 (Fig. 3C–F).

Taken together, these observations support the conclusion that nuclear HO-1 protects the endothelial cells against senescence, and that this protective effect does not rely on its antioxidant enzymatic activity.

### Repression of HO-1 nuclear translocation by SPP silencing exacerbated endothelial senescence

**Fig. 4:**
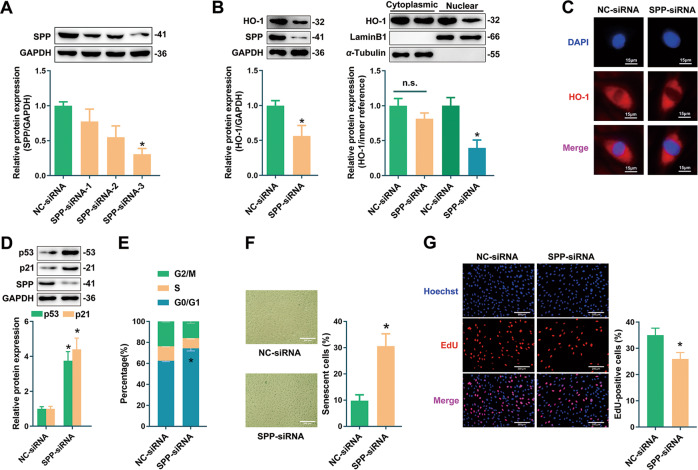
Reducing nuclear HO-1 by SPP knockdown exacerbated endothelial senescence. **A** Screening of SPP interference sequences by Western blot. Sequence-3 was used for the following experiments. ^*^P < 0.05 vs. NC-siRNA. *n* = 5. **B** Silencing of SPP decreased the expression of nuclear HO-1 rather than cytoplasmic HO-1. ^*^P < 0.05 vs. NC-siRNA. *n* = 5. **C** Silencing of SPP reduced fluorescence signal of nuclear HO-1. *n* = 3. **D** Silencing of SPP increased the expression of p53 or p21. ^*^P < 0.05 vs. NC-siRNA. *n* = 5. **E** Silencing of SPP increased the percentage cells at G0/G1 phase. ^*^P < 0.05 vs. NC-siRNA. *n* = 5. **F** Silencing of SPP increased the proportion of SA-β-gal staining cells. ^*^P < 0.05 vs. NC-siRNA. *n* = 5. **G** Silencing of SPP decreased the ratio of EdU-positive cells. ^*^P < 0.05 vs. NC-siRNA. *n* = 5.

In addition to upregulation of nuclear HO-1 through artificial modification, we also attempted to investigate the role of endogenous nuclear HO-1 by silencing SPP. As shown in Fig. 4A, siRNA-3 targeting SPP demonstrated the best efficiency and thus was used for the following experiments. SPP deficiency facilitated the reduction of total HO-1 which was primarily attributed to a decrease of nuclear HO-1 without altering its cytoplasmic expression, as implied by Western blot and immunofluorescence (Fig. 4B, C). Knockdown of SPP upregulated the expressions of cell cycle suppressors p53 and p21, induced cell cycle arrest at the G0/G1 phase, enhanced the proportion of SA-β-gal-positive cells, and decreased the number of proliferative cells (Fig. 4D–G). Therefore, these observations indicate that inhibition of SPP-mediated HO-1 nuclear trafficking by silencing SPP exacerbates endothelial senescence.

### Nuclear accumulation of HO-1 regulated protein binding rather than directly regulated aging genes

Next, we sought to explore the mechanisms underlying the anti-senescent effect of nuclear HO-1. RNA sequence analysis was used to screen the senescence-associated genes potentially regulated by nuclear HO-1. There were 52 up-regulated and 36 down-regulated differently expressed genes (DEGs) in the ΔHO-1 group, 166 up-regulated and 319 down-regulated DEGs in the Vector+Hemin group, as compared with Vector group (DEGs criteria: |fold change| ≥ 2, q value < 0.001) (Fig. S3A, B). *KEGG* signaling pathway analysis of DEGs was indicated in Fig. S3C, D. DNA damage has been recognized as a critical causal factor for the aging process [19]. Thus, we focus on not only “Aging” pathway, but also “Replication and repair” pathway. As shown in Fig. S3E, there were 52 common DEGs, but only 2 genes in “Replication and repair” and “Aging” pathways were found (Fig. S3E and F). Interestingly, *GO* pathway analysis showed that DEGs were strongly correlated to “binding” (molecular function), and 24 from 52 DEGs belonged to “protein binding” pathway (Fig. S4A–C). However, these 24 DEGs were lack of connection between each other based on STRING interaction network analysis (Fig. S4D). Therefore, it is most likely that nuclear HO-1 regulates protein binding rather than directly regulates aging genes.

### HO-1 interacted with NPM1 in the nucleus

**Fig. 5:**
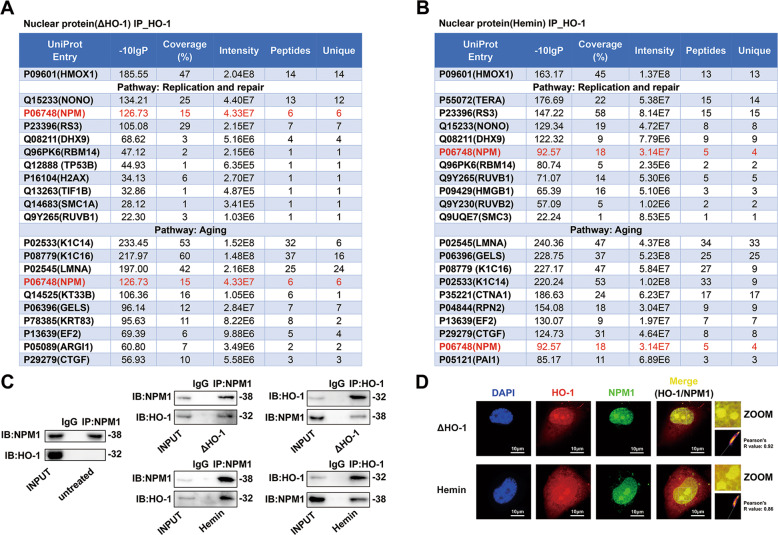
Nuclear HO-1 interacted with NPM1 in the nucleolus and nucleoplasm. **A** IP-MS screening analysis in “Aging” and “DNA replication and repair” pathways (HUVECs were transfected with ΔHO-1). **B** IP-MS screening analysis in “Aging” and “DNA replication and repair” pathways (HUVECs were stimulated by Hemin). **C** HO-1 interacted with NPM1 directly in HUVECs in the presence of ΔHO-1 or Hemin, but not in untreated cells. *n* = 3. **D** HO-1 colocalized with NPM1 in the nucleolus and nucleoplasm. *n* = 3. Colocalization 2D intensity histogram and Pearson’s R value were analysed by the Fiji’s plugin *Coloc 2*.

Since HO-1 is not a transcription factor, and no traditional DNA binding domain of HO-1 has been found [20], it seems unlikely that nuclear HO-1 regulates gene transcription directly. The hypothesized mechanism is that nuclear HO-1 interacts with other proteins through protein binding, and ultimately alters the function of those interacted proteins, similar as cytoplasmic HO-1 which regulated eNOS phosphorylation by interacting with eNOS and Akt [9]. In order to find out the key proteins interacted with nuclear HO-1, immunoprecipitation-mass spectrometry (IP-MS) analysis was performed to screen proteins interacted with nuclear HO-1 in HUVECs transfected with ΔHO-1 plasmid or treated with Hemin. We highlighted the top 10 proteins interacted with HO-1, screening from “Replication and repair” and “Aging” pathway through *KEGG* signaling pathway analysis. As shown in Fig. 5A, B, nucleophosmin (NPM1) demonstrated the highest frequency with a high mass spectrometry score in these pathways. The detected peptides of HO-1 and NPM1 were shown in Figs. S5 and S6. In addition, the interaction of HO-1 and NPM1 was confirmed by co-immunoprecipitation (co-IP) assay. In HUVECs transfected with ΔHO-1 or treated with Hemin, the physical interaction between HO-1 and NPM1 became obvious, as compared to that in the control cells (Fig. 5C). Moreover, immunofluorescence staining demonstrated that HO-1 and NPM1 colocalized in the nucleus, in particular the nucleolus (Fig. 5D). Since NPM1 is a key regulator of a myriad of biological functions, including aging [21], cell proliferation [22] and DNA repair [23], it is most likely that nuclear HO-1 inhibits endothelial senescence through interaction with NPM1.

### Nuclear HO-1 inhibited NPM1 nucleoplasm translocation and preserved p53-MDM2 interaction

**Fig. 6:**
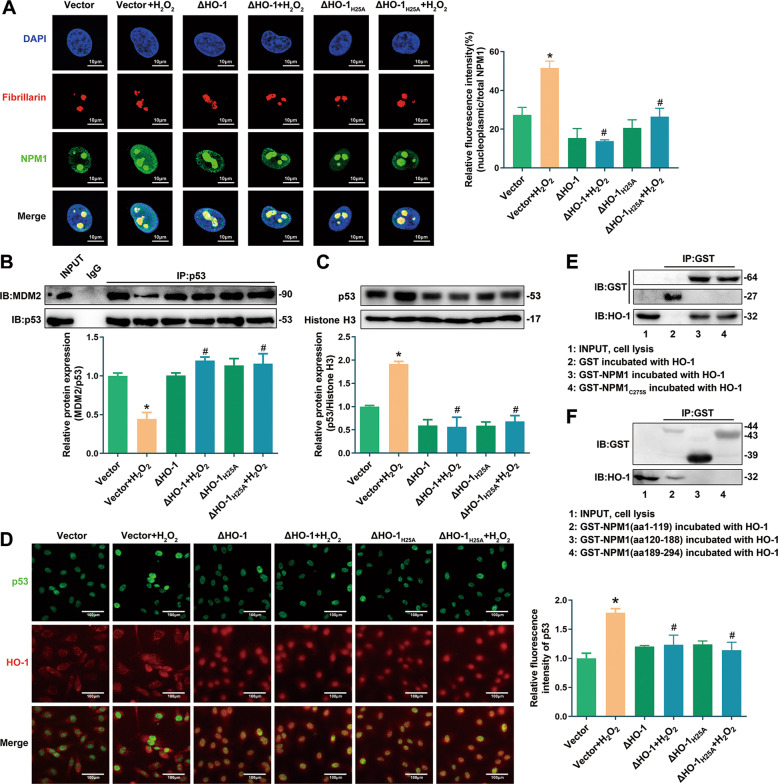
Nuclear HO-1 inhibited NPM1 translocation and p53 accumulation. **A** Transfection with ΔHO-1/ΔHO-1_H25A_ reversed the increasing fluorescence signal of nucleoplasmic NPM1 induced by H_2_O_2_. ^*^P < 0.05 vs. Vector; and ^#^*P* < 0.05 vs. Vector+H_2_O_2_. *n* = 3. Fibrillarin, the marker of nucleolus. **B** Transfection with ΔHO-1/ΔHO-1_H25A_ reversed the decreasing p53-MDM2 interaction induced by H_2_O_2_. ^*^P < 0.05 vs. Vector; and ^#^*P* < 0.05 vs. Vector+H_2_O_2_. *n* = 3. **C**, **D** Transfection with ΔHO-1/ΔHO-1_H25A_ reversed the up-regulation of nuclear p53 induced by H_2_O_2_. ^*^P < 0.05 vs. Vector; and ^#^*P* < 0.05 vs. Vector+H_2_O_2_. *n* = 3. **E** Identification of the interaction between HO-1 and GST-NPM1 or GST-NPM1_C275S_. *n* = 5. **F** Identification of the interaction between HO-1 and truncated NPM1 domains. *n* = 4.

NPM1 regulates aging and cell cycle via p53 [21]. Under stress, NPM1 is transported into nucleoplasm from nucleolus, and directly binds to p53, subsequently inhibits the degradation of p53 by E3 ubiquitin ligase MDM2 [24–26]. Indeed, transfection with ΔHO-1 or ΔHO-1_H25A_ prevented NPM1 nucleoplasm trafficking (Fig. 6A), repression of p53-MDM2 interaction (Fig. 6B), and up-regulation of nuclear p53 (Fig. 6C, D) induced by H_2_O_2_.

It has been reported that NPM1 translocation from nucleolus to nucleoplasm is triggered by glutathionylation at cysteine 275 [26]. However, HO-1 could interact with NPM1 C275S mutant, similar as the wild type NPM1, as implied by GST pull-down assay (Fig. 6E). These observations thus exclude the involvement of NPM1 glutathionylation in HO-1-NPM1 interaction.

**Fig. 7:**
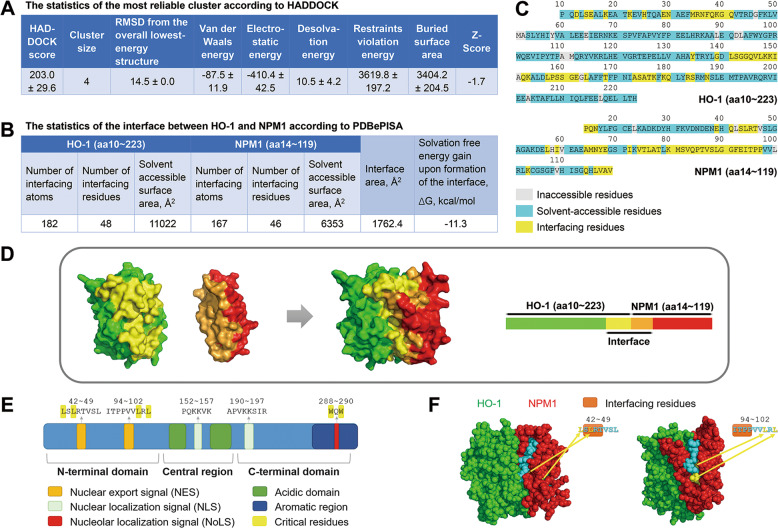
HO-1-NPM1 docking statistics and visual crystal structure. **A** Statistics of the top 1 cluster of HO-1-NPM1 complex according to HADDOCK. **B** Statistics of interface between HO-1 and N-terminal NPM1 according to PDBePISA. **C** Specific interfacing residues were demonstrated. **D** HO-1-NPM1 complex was visualized by PyMOL software. **E** Structure and functional domains of NPM1. **F** 3D structure information of interface between HO-1 and N-terminal NPM1.

Ulteriorly, the truncation experiments identified that nuclear HO-1 bound to the N-terminal region of NPM1 (aa1–119) (Fig. 6F). To further investigate how nuclear HO-1 influences NPM1 translocation, molecular docking of HO-1-NPM1 was conducted by HADDOCK methods [27] (Figs. 7A and S7A), and the interface residues of the best HO-1-NPM1 complex (cluster8_3) were analyzed by PDBePISA web server (Figs. 7B and S7B) [28]. There were 6 salt bridges and 14 hydrogen bonds in the interface (Fig. S7C, D). Besides, specific interface residues were visualized in Fig. 7C, D. Interestingly, N-terminal NPM1 contains two leucine-rich nuclear export signal (NES) motifs, including sequence 42–49 (leucines 42 and 44 are critical nuclear export residues) and sequence 94–102 (leucines 100 and 102 are critical nuclear export residues) (Fig. 7E) [29, 30]. Stereostructure of HO-1-NPM1 complex presented a steric hindrance around the NES motifs of NPM1 at the N-terminal domains (Fig. 7F). When HO-1 binds to NPM1, these critical nuclear export residues were masked (Fig. 7F), probably leading to suppression of NPM1 translocation. The results thus suggest that nuclear HO-1-NPM1 interaction weakened the nuclear export of NPM1 so that NPM1 translocation was inhibited, leading to the increase of p53-MDM2 interaction.

### HO-1-NPM1 interaction was pivotally involved in anti-senescent effect of nuclear HO-1

**Fig. 8:**
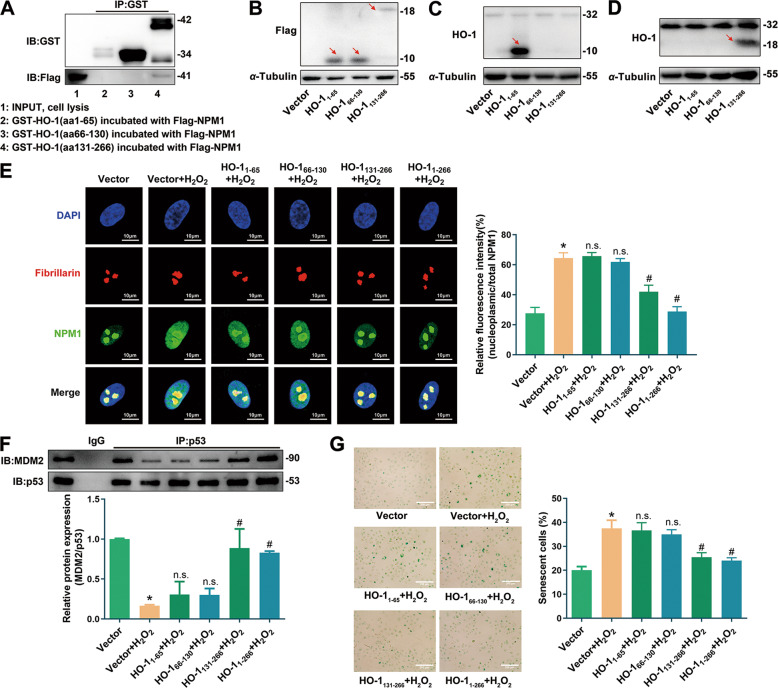
HO-1-NPM1 interaction reversed H_2_O_2_-induced NPM1 translocation and endothelial senescence. **A** Interaction between NPM1 and three truncated GST-HO-1 was tested by GST pulldown assay. *n* = 4. Detection of truncated HO-1 fragments with a Flag tag using (**B**) anti-Flag antibody; (**C**) anti-HO-1_aa1-30_ (Abcam, ab13248, corresponding to amino acids 1-30 of HO-1); and (**D**) anti-HO-1_C-terminus_ (Abcam, ab52947, corresponding to C-terminus of HO-1). Red arrow indicates the fragments of HO-1. *n* = 3. **E**, **F** Transfection with HO-1_131-266_ or HO-1_1-266_ instead of HO-1_1-65_ and HO-1_66-130_ reversed the increasing fluorescence signal of nucleoplasmic NPM1 and the decreasing p53-MDM2 interaction induced by H_2_O_2_. ^*^P < 0.05 vs. Vector; ^#^*P* < 0.05 vs. Vector+H_2_O_2_; and n.s. (non significant) vs. Vector+H_2_O_2_. *n* = 3. **G** Transfection with HO-1_131-266_ or HO-1_1-266_ instead of HO-1_1-65_ and HO-1_66-130_ reversed the increasing proportion of SA-β-gal-positive cells induced by H_2_O_2_. ^*^P < 0.05 vs. Vector; ^#^*P* < 0.05 vs. Vector+H_2_O_2_; and n.s. vs. Vector+H_2_O_2_. *n* = 5.

We tried to identify the interaction domain of nuclear HO-1 binding with NPM1. Three truncated HO-1 plasmids (aa1–65, 66–130, 131–266) were constructed according to the interface residues analysis of HO-1-NPM1 complex. GST pull-down assay showed that HO-1_131-266_, but not HO-1_1-65_ or HO-1_66-130_, could interact with NPM1 (Fig. 8A). Next, these HO-1 fragments with a Flag-tag were constructed and confirmed by antibodies to Flag, and to aa1–30 and C-terminus of HO-1 (Fig. 8B–D). The regulatory role of HO-1-NPM1 interaction in endothelial senescence was explored. As indicated by Fig. 8E and F, transfection with HO-1_131-266_ or HO-1_1-266_, instead of HO-1_1-65_ or HO-1_66-130_, prevented NPM1 nucleoplasm trafficking and repression of p53-MDM2 interaction induced by H_2_O_2_. In addition, SA-β-gal staining assay intuitively showed that the anti-senescent effect of nuclear HO-1 was abolished when HO-1 (aa131-266) was absent (Fig. 8G). These results suggest that interaction of HO-1_131-266_ with NPM1 inhibits NPM1 translocation and preserves p53-MDM2 interaction, finally protects against stress-induced endothelial senescence.

## Discussion

Endothelial senescence is a hallmark of majority of cardiovascular diseases, such as atherosclerosis, hypertension and heart failure [6–8]. The present study reveals a novel mechanism by which HO-1 protects against endothelial senescence after it is transported into the nucleus of endothelial cells. The nuclear HO-1 confers protection through interaction with NPM1, leading to disruption of p53/NPM1/MDM2 complex, finally facilitating p53 degradation by MDM2.

Although HO-1 is commonly known as an antioxidant enzyme located in the cytoplasm, accumulating evidences have shown that HO-1 undergoes proteolytical cleavage from ER and translocates to other organelles including caveolae [31], mitochondria [32] and nucleus [20] in response to stress stimuli. HO-1 nuclear localization has been detected in a few cell types [33–38]. Nuclear HO-1 is reported to participate in the regulation of various cellular functions, such as oxidative stress [20], inflammation [37], DNA repair [39], virus infection [40] and tumor progression [15, 41]. Our study demonstrated that HO-1 accumulated in the nuclei of senescent endothelial cells induced by H_2_O_2_, Ang II, ox-LDL and D-gal, in which HO-1 was upregulated (Fig. 1A, B). Likewise, nuclear accumulation of HO-1 was observed in HUVECs treated with Hemin, a pharmacological inducer which could ameliorate endothelial senescence (Fig. 1C, D). In the in vivo endothelial senescence model induced by ischemic stress, the total expression of HO-1 was also augmented (Fig. 1E). However, HO-1 induction and nuclear accumulation was observed in stress-induced endothelial senescence rather than replicative senescence (Fig. S2). Indeed, up-regulation of HO-1 has been reported in stress-induced conditions such as ischemia/reperfusion injury, atherosclerosis, hypertension, and heart failure [42–44]. By contrast, age-related decrease in HO-1 expression has been well documented in various organs, including brain [45], carotid bodies [46] and heart [47, 48]. Taken together, these observations suggest that HO-1 expression and nuclear accumulation are induced in endothelial senescence under stress.

It is still unclear how HO-1 is transported into the nucleus. Generally, nuclear trafficking of HO-1 includes at least two steps: (1) the C-terminal TMS of HO-1 is cleaved; and (2) the truncated HO-1 shuttles into the nucleus. It has been reported that SPP is responsible for enzymatic cleavage of the TMS of HO-1 [15]. Our observations that SPP expression was significantly augmented in HUVECs treated with H_2_O_2_ or Hemin (Fig. 1F and G), and that SPP knockdown inhibited the nuclear accumulation of HO-1 (Fig. 4A–C), might suggest that HO-1 nuclear trafficking relies on the upregulation of SPP. After cleavage by SPP, the truncated HO-1 might possibly diffuse freely through the nuclear pore complex, since it is a small protein with the molecular weight less than 50 kDa [49–51]. However, the fact that HO-1 tends to shuttle into nucleus under stress or hypoxia [20], might probably exclude the possibility that HO-1 nuclear localization is a random event through diffusion. Another explanation is that HO-1 exposes its NLS following cleavage by SPP. Currently, no classical NLS on HO-1 was reported, nor was detected by NLS prediction software such as cNLS Mapper and PSOR II Prediction. However, a “nuclear shuttling sequence (NSS)” (amino acids 207–221) of HO-1 has been reported to participate in the nuclear accumulation of HO-1 [20]. Nevertheless, the present observations did not allow further speculation on the exact mechanism underlying HO-1 nuclear transport.

Our findings prompt the conclusion that nuclear HO-1 ameliorates endothelial senescence. This is based on the following observations: (1) overexpression of ΔHO-1, a truncated HO-1 without TMS which was found to accumulate in the nucleus, inhibited H_2_O_2_-induced endothelial senescence (Fig. 2); (2) overexpression of nuclear HO-1 by infection of Ad-ΔHO-1(3NLS) could still preserve the inhibitory effect against endothelial senescence even though endogenous HO-1 was knocked down by CRISPR/Cas9 (Fig. 3); and (3) repression of HO-1 nuclear translocation by silencing of SPP exacerbated endothelial senescence (Fig. 4). Considering that nuclear HO-1 was upregulated in the stress-induced senescent endothelial cells, nuclear HO-1 might probably act as a compensatory role in protecting cells from aging stress. However, this endogenous compensation may be limited, judging from observations that HO-1 was downregulated in replicative endothelial senescence and in the endothelium of aging animals (Fig. S2). Thus, strategies targeting induction of HO-1 nuclear accumulation might suggest therapeutic potential in endothelial senescence associated diseases. Moreover, upregulation of nuclear HO-1 in Hemin-treated cells might also suggest that nuclear HO-1 at least partially contributes to the anti-senescent effect of Hemin. Most interestingly, the protective effect of nuclear HO-1 does not rely on its antioxidant catalytic capability, as implied by the observations that overexpression of ΔHO-1_H25A_, the catalytically inactive form of ΔHO-1, exhibited anti-senescent effect. These findings are in line with previous reports that nuclear HO-1 regulates cellular functions independent of its catalytic activity [15, 20, 37, 39, 41].

The present study attempted to find out the regulatory mechanism of nuclear HO-1 in endothelial senescence. HO-1 is not a transcription factor, and there is no DNA binding domain in HO-1 structure, it seems impossible that nuclear HO-1 directly regulates the transcription of DEGs. Indeed, according to RNA sequence analysis, nuclear accumulation of HO-1 might regulate many genes associated with protein binding rather than aging process (Figs. S3 and S4). It is hypothesized that nuclear HO-1 may act as an adapter protein that regulates protein-protein interactions, thereby interacting with regulators of senescence in the nucleus, finally altering the function of these regulators. Intriguingly, the IP-MS, co-IP, immunofluorescence and GST pull-down assay results confirmed that there was physical interaction between nuclear HO-1 and NPM1 (Figs. 5 and 6E). NPM1, also known as B23, is an abundant nucleolar phosphoprotein, mainly participating in ribosome biogenesis, genomic stability maintenance, p53-dependent stress response and growth modulation [52, 53]. The regulation of p53 by NPM1 plays a pivotal role in cell cycle control. When NPM1 shuttles from nucleolus to nucleoplasm under stress, it directly binds to p53 or MDM2, thus disrupting p53/MDM2 interaction and preventing the degradation of p53 by MDM2, finally leading to cell growth arrest [21, 24, 25]. In addition, NPM1 can sequester Arf into the nucleolus and enhance the interaction between Arf and MDM2, thereby suppressing p53/MDM2 interaction and leading to the activation of p53 in the nucleoplasm [22, 54–56]. The present study suggests that physical interaction of nuclear HO-1 and NPM1 represses NPM1 function in a p53-dependent manner. This is based on the observations that NPM1 was sequestered in the nucleolus by nuclear HO-1, and that p53/MDM2 interactions were enhanced and p53 expression was decreased after overexpressing nuclear HO-1 (Figs. 6A–D, 2). It is most likely that nuclear HO-1 prevents NPM1 trafficking from nucleolus to nucleoplasm, abolishing the interactions of NPM1/p53/MDM2 in the nucleoplasm, therefore preventing p53 activation by NPM1, subsequently resisting cell cycle arrest. It is reported that NPM1 translocation is regulated by post-translational modifications such as acetylation, phosphorylation and glutathionylation [26, 57, 58]. Among all, S-glutathionylation of NPM1 on Cys275 is important for NPM1 nucleoplasmic trafficking and p53 activation under oxidative stress [26]. However, our results eliminated the involvement of NPM1 Cys275 S-glutathionylation in effect of nuclear HO-1, since interaction between nuclear HO-1 and NPM1 was persistent after Cys275 of NPM1 was mutated (Fig. 6E).

We further explored the interaction mode between nuclear HO-1 and NPM1. The GST pull-down assay results showed that nuclear HO-1 only interacted with truncation of NPM1 N-terminal portion (Fig. 6F). Molecular docking and PDBePISA interface assay provided specific interfacing residues of HO-1-N-terminal NPM1 complex (Fig. 7A–D). NPM1 structural architecture is characterized by two NESs (aa42–49, aa94–102), a bipartite NLS (aa152–157) and a nucleolar localization signal (NoLS, aa288–290) (Fig. 7E). Under normal physiological conditions, the mask of NESs, together with the exposure of NLS and NoLS, mediate the anchoring of NPM1 into nucleolus [59–61]. According to the molecular docking results, a steric hindrance of HO-1-NPM1 complex around NES of N-terminal NPM1 was identified, suggesting that HO-1-NPM1 interaction might mask the critical nuclear export residues of NPM1 (Fig. 7F). Unfortunately, it is unavailable to determine whether or not HO-1-NPM1 interaction affects the exposure of NLS and NoLS which located at the central region and C-terminal region of NPM1, due to the lack of crystal structure of complete NPM1. Taken into considerations that nuclear HO-1 prevented nucleoplasmic translocation of NPM1 induced by H_2_O_2_ (Fig. 6A), it is possible that HO-1-NPM1 interaction might not only mask the NESs, but also expose the NLS and NoLS of NPM1 to sequester NPM1 into the nucleolus. Moreover, HO-1_131-266_ was identified as the interaction domain of nuclear HO-1 binding with NPM1, as implied by the interface residues analysis of HO-1-NPM1 complex and GST pull-down assay results (Figs. 7 and 8A). HO-1_131-266_, but not HO-1_1-65_ or HO-1_66-130_, inhibited H_2_O_2_-induced NPM1 nucleoplasm translocation, suppressed p53-MDM2 interaction, and subsequently ameliorated stress-induced endothelial senescence (Fig. 8). These observations support the conclusion that nuclear HO-1-NPM1 interaction is pivotally involved in the anti-senescent effect of nuclear HO-1.

In conclusion, the present study provides novel insight into the regulatory role of HO-1 in endothelial senescence and vascular homeostasis. HO-1 is accumulated in the nucleus in stress-induced senescent endothelial cells and confers protection against endothelial senescence. Mechanistically, nuclear HO-1 interacts with NPM1 N-terminal portion, prevents NPM1 translocation from nucleolus to nucleoplasm, thus disrupts NPM1/p53/MDM2 interactions and inhibits p53 activation by NPM1, finally resists stress-induced endothelial senescence. The protective effect of nuclear HO-1 is not dependent on its antioxidant enzymatic activity. Thus, these findings expand our understanding of HO-1 as a promising therapeutic strategy for vascular senescence-related cardiovascular diseases.

## Supplementary information

Supplementary Figure Legends

Figure S1

Figure S2

Figure S3

Figure S4

Figure S5

Figure S6

Figure S7

## Data Availability

The datasets used and/or analyzed during the current study are available from the corresponding author on reasonable request.
