# Supplementary material for: HO-1 nuclear accumulation and interaction with NPM1 protect against stress-induced endothelial senescence independent of its enzymatic activity
Source: Cell Death Dis. 2021 Jul 26;12(8):738. doi: 10.1038/s41419-021-04035-6 (PMC8313700; doi:10.1038/s41419-021-04035-6)
Supplement: Supplementary file 1 — Supplementary Figure Legends [file 41419_2021_4035_MOESM1_ESM.docx]

**Fig. S1. Endothelial senescence was detected in the mice with partial carotid ligation.**

(A) Schematic diagram of ligation, ECA (external carotid artery), ICA (internal carotid artery), OA (occipital artery), STA (superior thyroid artery), LCA (left common carotid artery), RCA (right common carotid artery), RSA (right subclavian artery), LSA (left subclavian artery), red arrow indicates blood flow direction. (B) Ultrasound showed blood vessels narrowed and blood flow reduced. (C) The percentage of senescent endothelial cells increased in LCA with ligation, ^*^P < 0.05 vs. RCA without ligation. n = 4. Blue arrow indicates SA-β-gal-positive cell.

**Fig. S2. The expression of HO-1 decreased in replicative endothelial senescence.**

(A) The expression of the total, cytoplasmic and nuclear HO-1 decreased in the senescent HUVECs (Passage 12) as compared with the young cells (Passage 3). ^*^P < 0.05 vs. P3. n = 5. (B) The percentage of senescent endothelial cells increased in aorta of 23-month-old mice, ^*^P < 0.05 vs. 2 months. n=8. Blue arrow indicates SA-β-gal-positive cell. (C) The level of HO-1 decreased in aorta of 23-month-old mice, ^*^P < 0.05 vs. 2 months. n=4.

**Fig. S3. *KEGG* Pathways assay of DEGs detected by RNA sequence analysis in HUVECs treated with Hemin or transfected with ΔHO-1.**

(A&B) DEGs were shown by volcano plot. (C) RNA-sequence analysis in *KEGG* Pathways (Vector v.s. ΔHO-1). (D) RNA sequence analysis in *KEGG* Pathways (Vector v.s. Vector+Hemin). (E&F) Details of common DEGs.

**Fig. S4. *GO* Pathways and STRING interaction network analysis of DEGs**

(A) *GO* Pathways analysis (Vector v.s. ΔHO-1). (B) *GO* Pathways analysis (Vector v.s. Vector+Hemin). (C&D) Details and STRING interaction network analysis of DEGs belong to GO:0005515.

**Fig. S5. Peptides of HO-1 and NPM1 detected by IP-MS (Part 1).**

Specific peptides of HO-1 and NPM1 were analysed by IP-MS (HUVECs were transfected with ΔHO-1).

**Fig. S6. Peptides of HO-1 and NPM1 detected by IP-MS (Part 2).**

Specific peptides of HO-1 and NPM1 were analysed by IP-MS (HUVECs were stimulated by Hemin).

**Fig. S7. Screening of the best HO-1-NPM1 complex according to HADDOCK and PDBePISA.**

(A) The top cluster (cluster 8) was the most reliable according to HADDOCK. (B) The best 4 protein complexes in cluster 8. (C) Interface salt bridges of cluster8_3. (D) Interface hydrogen bonds of cluster8_3.
