# Supplementary figures and images for: HO-1 nuclear accumulation and interaction with NPM1 protect against stress-induced endothelial senescence independent of its enzymatic activity
Source: Cell Death Dis. 2021 Jul 26;12(8):738. doi: 10.1038/s41419-021-04035-6 (PMC8313700; doi:10.1038/s41419-021-04035-6)

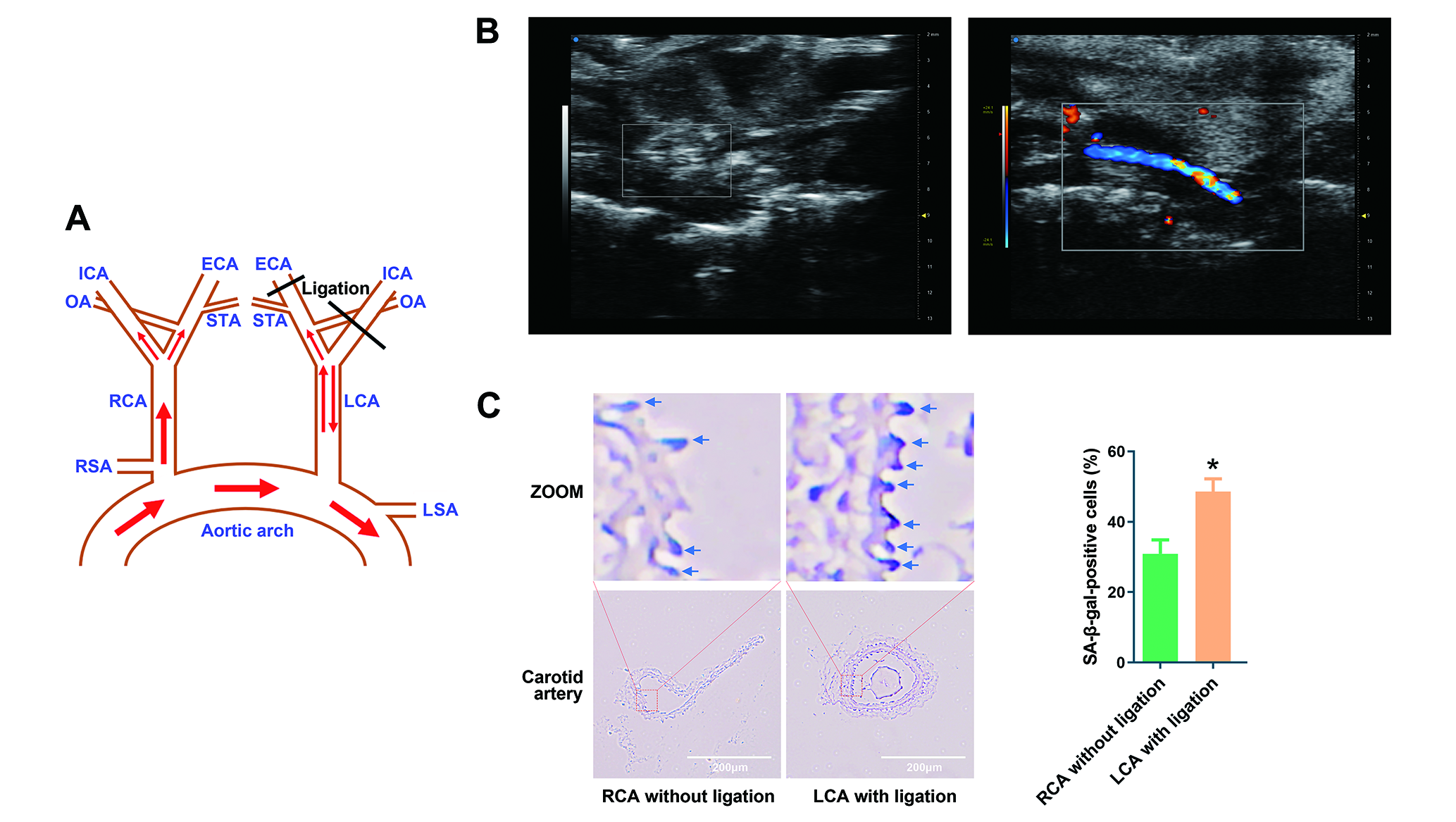

Supplement: Supplementary file 2 — Figure S1 [file 41419_2021_4035_MOESM2_ESM.tif]

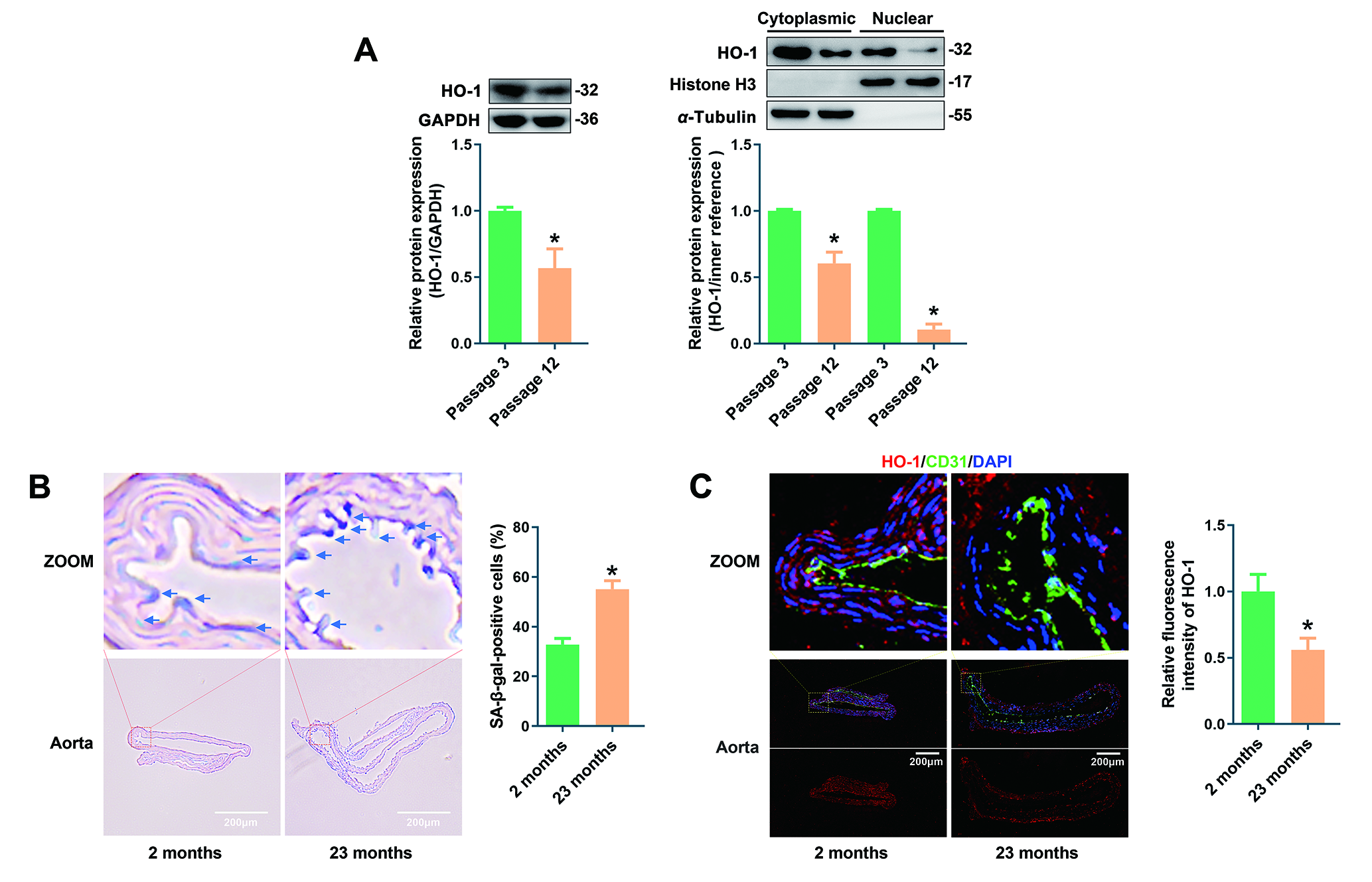

Supplement: Supplementary file 3 — Figure S2 [file 41419_2021_4035_MOESM3_ESM.tif]

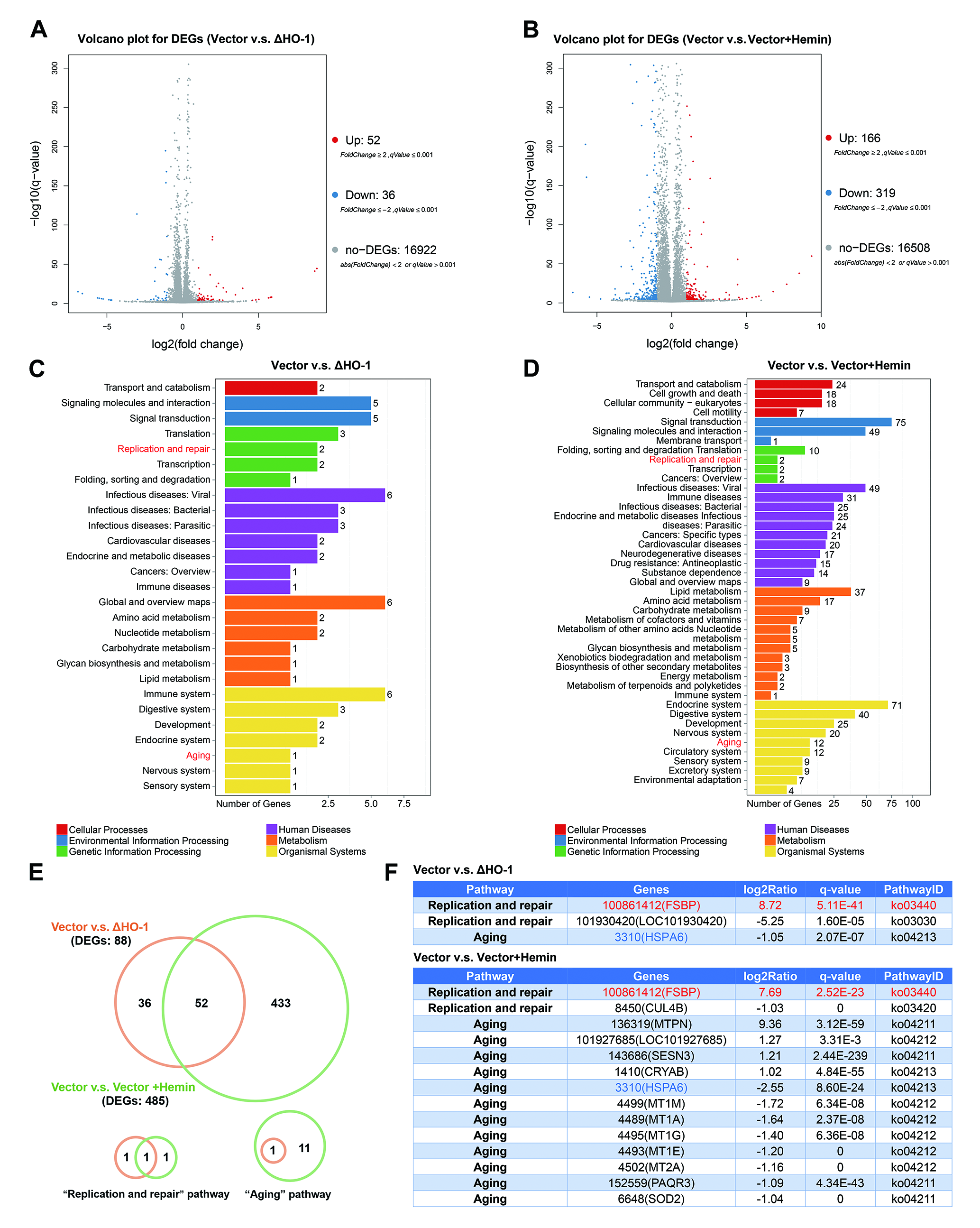

Supplement: Supplementary file 4 — Figure S3 [file 41419_2021_4035_MOESM4_ESM.tif]

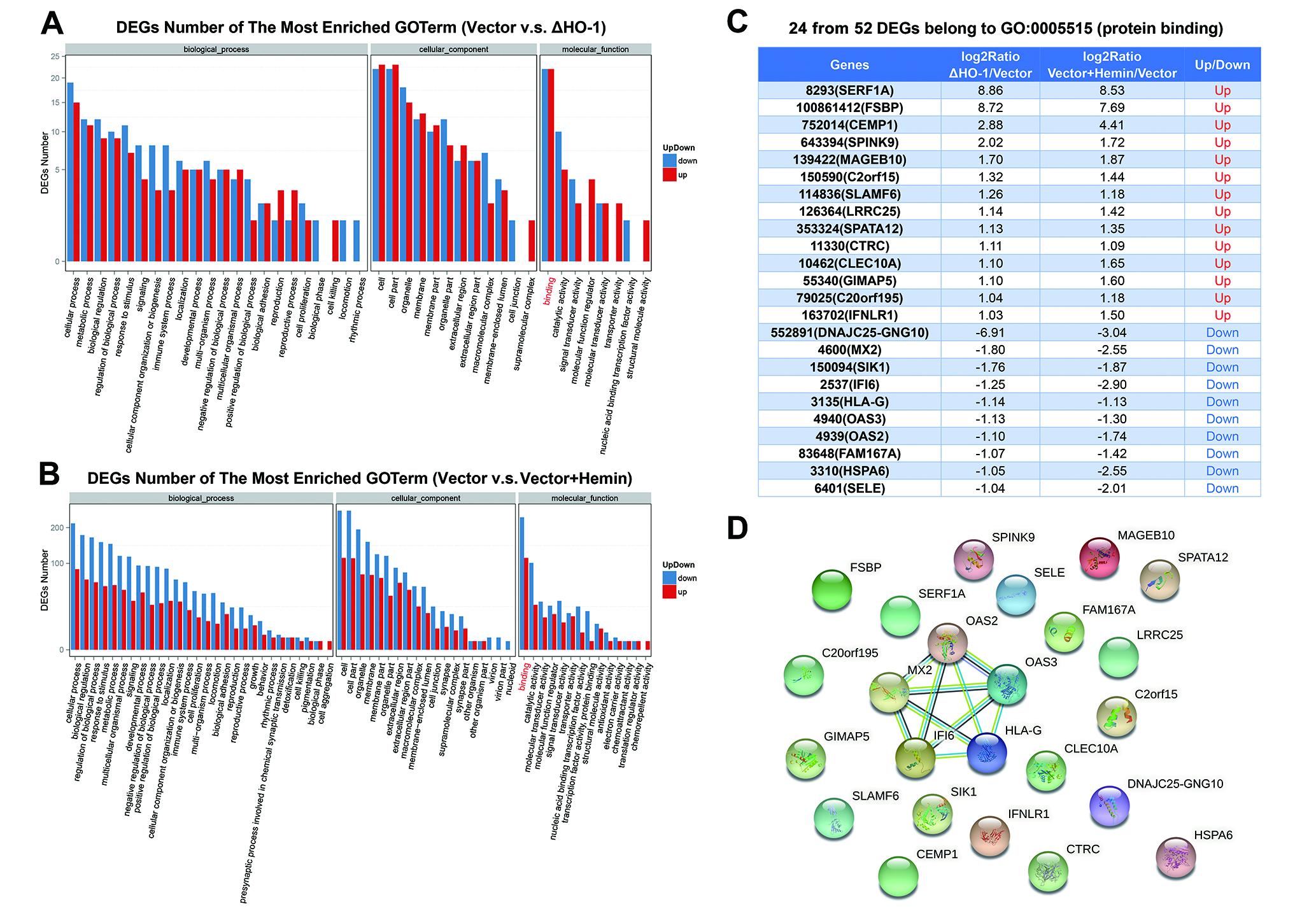

Supplement: Supplementary file 5 — Figure S4 [file 41419_2021_4035_MOESM5_ESM.tif]

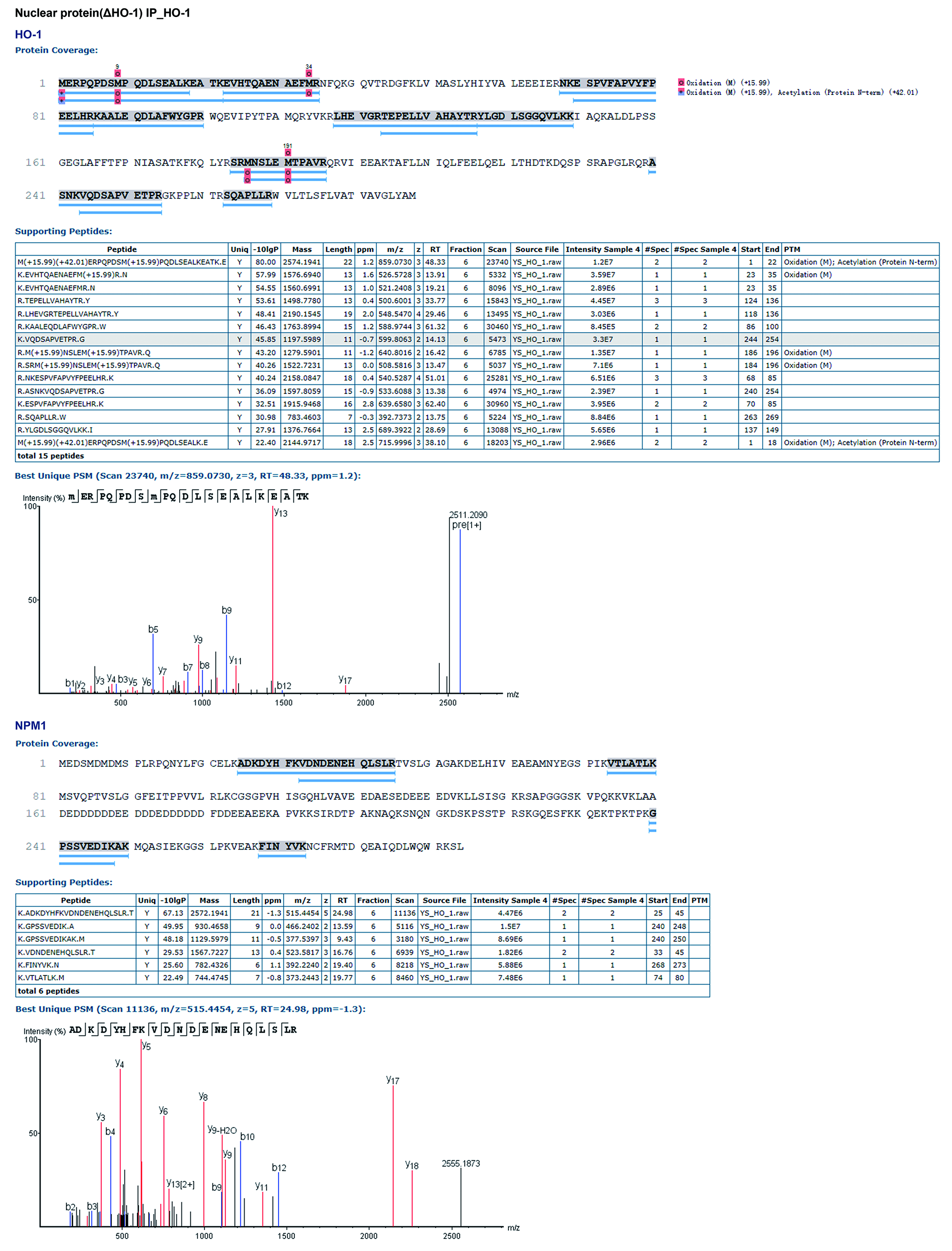

Supplement: Supplementary file 6 — Figure S5 [file 41419_2021_4035_MOESM6_ESM.tif]

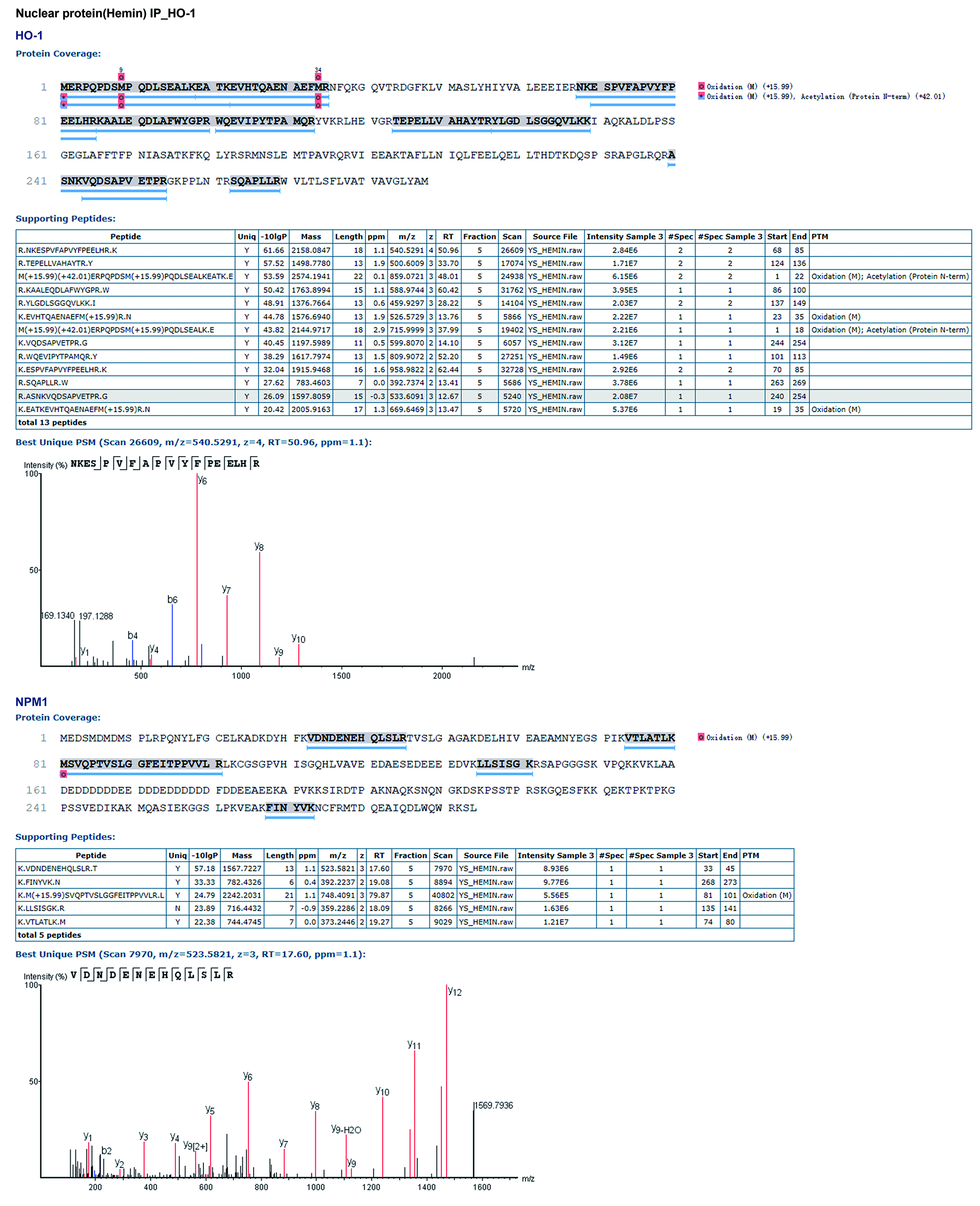

Supplement: Supplementary file 7 — Figure S6 [file 41419_2021_4035_MOESM7_ESM.tif]

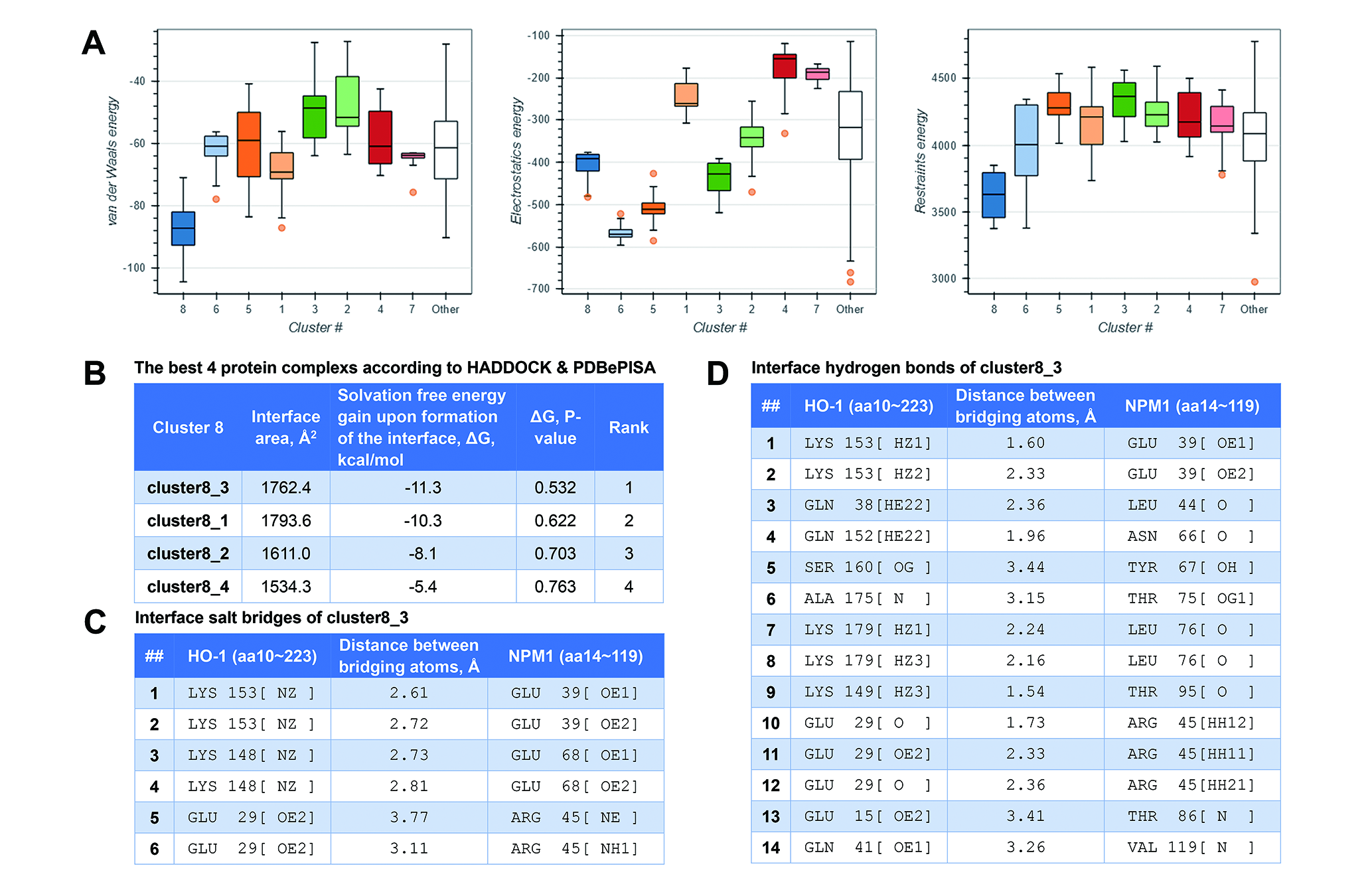

Supplement: Supplementary file 8 — Figure S7 [file 41419_2021_4035_MOESM8_ESM.tif]
